# Ecological Risks of Zinc Oxide Nanoparticles for Early Life Stages of Obscure Puffer (*Takifugu obscurus*)

**DOI:** 10.3390/toxics12010048

**Published:** 2024-01-08

**Authors:** Shengkai Tang, Jun Wang, Xuexia Zhu, Dongdong Shen

**Affiliations:** 1 Freshwater Fisheries Research Institute of Jiangsu Province, Nanjing 210017, China; 13372015836@163.com; 2 College of Animal Science and Technology, Yangzhou University, Yangzhou 225009, China; junwang1990@yzu.edu.cn (J.W.); 008410@yzu.edu.cn (X.Z.)

**Keywords:** biochemical analyses, early life stage, survival, *Takifugu obscurus*, ZnO nanoparticle

## Abstract

Nanoparticles of zinc oxide (ZnO NPs) are extensively used in various applications, and their widespread use leads to their environmental presence, particularly in wastewater treatment plant effluents, rivers, and soil. This study focuses on the obscure puffer, *Takifugu obscurus*, an economically important fish in China, aiming to assess the toxic effects of ZnO NPs on its early life stages, emphasizing the need for understanding the ecological implications of ZnO NP exposure in aquatic environments. Exposure during the hatching stage resulted in a significant decrease in hatching rates, with embryos displaying surface coating at higher ZnO NP concentrations. Newly hatched larvae experienced deformities, and post-hatching exposure led to pronounced reductions in survival rates, particularly with higher ZnO NP concentrations. Two-month-old juveniles exposed to increasing ZnO NP concentrations exhibited a consistent decline in survival rates, emphasizing concentration-dependent adverse effects. Biochemical analyses revealed elevated malondialdehyde (MDA) levels and decreased glutathione (GSH), superoxide dismutase (SOD), and catalase (CAT) activities in various tissues, indicating oxidative stress. This study underscores the ecological risks of ZnO NP contamination in aquatic environments, emphasizing the need for careful consideration of nanoparticle exposure in aquatic ecosystems.

## 1. Introduction

Nanoparticles of zinc oxide (ZnO NPs) are currently some of the most widely used engineered nanomaterials. They are extensively employed in cosmetics, disinfectants, paints, and other applications. In the field of coatings, nanoscale zinc oxide accounts for 17% of the total usage, second only to the rubber industry [1]. ZnO is employed as a prominent photocatalyst for the removal of persistent organic pollutants (POPs) in water and air due to its eco-friendly and efficient properties [2]. Additionally, its bactericidal and bacteriostatic properties, stemming from the generation of reactive oxygen species (ROS), lead to its application in food packaging [3]. Due to their universal use, nanomaterials inevitably enter the environment (atmosphere, water bodies, soil, etc.) during production, storage, transportation, and use. ZnO NPs have already been found to be widely present in the environment, particularly in wastewater treatment plant effluents and sludge, rivers, and soil. Chauque et al. found that even after wastewater treatment, effluent still contains low concentrations of ZnO NPs, approximately 50–200 μg L^−1^, while the concentration in sludge can be much higher, reaching approximately 3000 mg kg^−1^ [4]. In this situation, it has been proposed that the majority of released ZnO nanoparticles will likely accumulate in sludge, reaching an estimated concentration of ≈24 µg g^−1^. This sludge is then typically incorporated into soils as biosolids or fertilizer; additionally, a portion of the particles may be released into freshwater environments through effluents [5].

Because of their small size compared to conventional particles and their large surface area, NPs possess greater surface activity and mobility, which may lead to more significant harmful biological effects on living cells and pose a greater threat to natural environments [6]. The toxic mechanisms of ZnO NPs are closely related to the characteristics of the particles and their interaction with biological entities. Generally considered from the perspective of particle–cell interactions, there are three main toxic mechanisms attributed to ZnO NPs in water: (1) the gradually release of zinc ions and dissolution, especially under oxidizing conditions; (2) the generation of ROS under light conditions because of the photocatalytic properties of ZnO NPs; and (3) direct contact with cells and potentially blocking various transport channels on the cell membrane, leading to changes in membrane permeability and resulting in cell rupture [7,8,9]. Franklin et al. found that ZnO NPs, bulk ZnO powder, and ZnCl_2_ had similar toxic effects on freshwater microalga (*Pseudokrichneriella subcapitata)*, with the toxicity primarily attributed to the dissolution of zinc ions, as evidenced by comparable 72 h LC_50_ values close to 60 μg Zn L^−1^ [10]. However, Zhang et al. observed that the toxicity of ZnO NPs to the bacteria *Escherichia coli* increased with prolonged storage durations, with 1-day storage requiring a concentration of 10 g L^−1^ for 100% mortality, while 90-day and 120-day storage achieved 100% mortality at 4 g L^−1^ [11]; additionally, exposure to light significantly enhanced toxicity, suggesting that extended storage under light conditions resulted in a greater release of reactive oxygen species and increased toxicity. Additionally, the toxicity of ZnO NPs to fish is higher than that of the equivalent concentration of a zinc ion solution, indicating that ion toxicity is only a part of nanoparticle toxicity [12].

A study in the journal Science underscored the imperative to intensify research efforts concerning the toxicity of nanoscale materials [13]. Simultaneously, a corresponding editorial article in Nature advocated for the exploration of the biological toxicity implications associated with nanoscale materials and nanotechnology [14]. Following this, the study of the biological toxicity of synthetic nanomaterials has become a prominent focus of global research, with a rapid growth in the past decade in research dedicated to assessing the ecotoxicological effects of nanoparticles on aquatic organisms. The potential for the bioaccumulation and biomagnification of ZnO NPs in aquatic environments highlights the importance of studying their ecotoxicological effects on various organisms, as well as their long-term impact on aquatic ecosystems [15,16]. Aquatic vertebrates, such as fish, may come into contact with ZnO NPs through multiple pathways encompassing both waterborne and food-borne exposure, which involves the ingestion of prey or organisms that previously accumulated ZnO NPs in their environment. In particular, the effects of ZnO NPs on fish can vary depending on the developmental stage of the fish. ZnO NPs caused developmental abnormalities in zebrafish, including tail edema, pericardial edema, and yolk sac edema [17]. In adult crucian carp (*Carassius carassius*), ZnO NPs induced immune toxicity through the release of neutrophil extracellular traps (NETs) and oxidative stress while also exerting toxic effects on the liver [18]. A concentration of 1 mg L^−1^ of ZnO NPs could significantly reduce the hatching rate of zebrafish [19]. Recent studies demonstrated that ZnO NPs have varying toxic effects on different developmental stages of fish, including developmental abnormalities, oxidative stress, DNA damage, impaired growth, and even mortality. Therefore, a comparative investigation into the impact of ZnO NPs on various developmental stages of fish is necessary. ROS production is recognized as a potential mechanism underlying the toxicity induced by NPs, a phenomenon substantiated in previous studies that elucidated its role in eliciting oxidative stress [12,20]. Furthermore, ROS can initiate oxidative damage to DNA, proteins, lipids, and various organelles [21]. Typically, living organisms employ antioxidant enzymes like superoxide dismutase (SOD), glutathione peroxidase (GPx), and catalase (CAT) to counteract oxidative-stress-induced damage. Malondialdehyde (MDA), a widely recognized marker of lipid peroxidation, holds significant predictive value in numerous studies, underscoring its status as a molecular mechanism behind NP-induced toxicity. Consequently, these indicators serve as crucial parameters for monitoring and identifying environmental pollutants.

Here, we studied the obscure puffer, *Takifugu obscurus*, one of the major anadromous migratory fish in the Yangtze River, China, and an economically important fish species in China and East Asian countries. The early life stage of *T. obscurus* is a period of rapid development and sensitivity to environmental changes, and it is the most promising stage for detecting an effect. Six concentrations (0, 10, 20, 40, 60, and 80 mg L^−1^) of ZnO nanoparticles were selected, taking into consideration both the toxic effects of ZnO NPs on fish and the concentrations found in natural water bodies. This study aimed to investigate the toxic effects of ZnO NPs on *T. obscurus* individuals in early life stages, including embryos, early hatched larvae, and juveniles.

## 2. Methods and Materials

### 2.1. Embryos, Newly-Hatched Larvae, and Juveniles of T. obscurus

All experiments in this study were conducted in compliance with the guidelines and regulations set forth by the Institutional Animal Care and Use Committee (IACUC) and were performed at Nanjing Normal University in Nanjing, China, under the approved protocol (permit number SYXK2015-0028).

The artificial insemination of *T. obscurus* was conducted in a fish farm in Yangzhong City, Jiangsu Province, China. To obtain fertilized eggs, we followed the method outlined by Yang and Chen [22] wherein a luteinizing-hormone-releasing hormone analogue (LHR-A1) was administered to 3 male and 1 female adult fish twice. Subsequently, the fertilized eggs were immediately transferred to our lab within two hours. The fertilized eggs were subsequently placed into tanks located within an incubator chamber set at a temperature of 23 ± 1 °C, maintaining a pH level of 7.1 ± 0.2 and ensuring continuous aeration. After 16 h, any damaged or deceased fertilized eggs were removed using wide-mouth pipettes. A portion of healthy eggs were set aside for subsequent embryo-related experiments, and the rest were gently agitated with feathers to ensure even distribution. The remaining eggs were utilized in the hatching process to obtain larvae. Healthy newly hatched larvae were randomly selected for further experiments approximately 24 h after hatching.

Two-month-old *T. obscurus* juveniles, measuring approximately 5.93 ± 1.12 cm in length and weighing around 17.46 ± 2.21 g, were procured from the fish farm. Subsequently, they were transported to the laboratory and temporarily reared in a 100 L tank with dissolved oxygen levels above 6.9 mg L^−1^, a light-to-dark cycle of 12 h each, a pH of 7.1 ± 0.3, and a temperature of 23 ± 1 °C. The juveniles were fed twice daily with commercial fish food. To ensure water quality, the lower half of the aquarium water was replaced daily. After 10 days, healthy individuals were randomly selected for the assessment of the effects of ZnO NPs on their survival and biochemical responses.

### 2.2. Preparation of Nanomaterials and Reagents

ZnO nanoparticles (diameter = 50 nm) were obtained from Nanjing Haitai Nanoparticles, Ltd. (Nanjing, China). To prepare a stock solution of 0.5 g L^−1^ ZnO NPs for the experiment, the purchased ZnO NPs were dissolved in Milli-Q water and subjected to 30 min of sonication in an ice water bath at 40 kHz. Subsequently, this stock solution was further diluted to obtain six different concentrations (0, 10, 20, 40, 60, 80 mg L^−1^). Diagnostic reagent kits supplied by the Nanjing Jiancheng Bioengineering Engineering Research Institute of China (Nanjing, China) were used to assess the levels of MDA, CAT, SOD, NKA, and glutathione (GSH).

### 2.3. Measurements of Total Dissolved Zn Contents

Characterization of ZnO NPs was conducted in the absence of fish. The size of the ZnO NPs and the Zeta potential of the media at various time intervals (0, 1, 6, 12, and 24 h) were determined using the Z3000 Nicomp Dynamic Light Scattering (DLS) technique (PSS, Orlando, FL, USA). For the assessment of the total zinc content in each experimental medium after 24 h of exposure, we employed the PE ICAP 6300 ICP-MS (Thermo Fisher Scientific, Waltham, MA, USA)(inductively coupled plasma mass spectrometer) method, as outlined in [23]. Since the test solutions were refreshed daily throughout the experiment, real-time total zinc concentrations in the absence of fish or embryos, encompassing both Zn particles and dissolved Zn ions over 24 h, were measured. After the 24 h period, uniform 10 mL aliquots of each test solution were collected for analysis.

### 2.4. Experimental Design

The experiments for embryos and newly hatched larvae were conducted using 100 mL beakers containing 80 mL of media. Each beaker was used to rear 40 embryos or 20 newly hatched larvae. The two-month-old juveniles were placed in a 48 cm × 34 cm × 27 cm aquarium containing 30 L of media. Each treatment was replicated three times. To maintain consistency, test solutions were replenished daily, and fresh ZnO NP stock solutions were prepared daily as well. Observations of the experimental progress were made daily, and any moldy and whitish embryo or deceased larva or juvenile were promptly removed. The embryos were delicately rinsed twice with a rubber-tip dropper to mitigate the potential for substance accumulation before they were transferred to fresh test solutions.

Biochemical tests were conducted on juveniles after 96 h of exposure to two different ZnO NP concentrations (0, 40 mg L^−1^) in three tissues: the gill, kidney, and intestine. A lethal dose of MS−222 neutralized using NaOH was then employed to euthanize the fish [24]. Specimens of the gills, kidney, and liver of the juveniles were obtained, and cold physiological saline (0.6% NaCl) was used to rinse them. The specimens were then transferred into 2 mL of 0.7% normal saline. Subsequently, centrifugation was carried out at 4000× *g* for 10 min at 4 °C to remove any homogenate fragments, including cartilage and cells. All resulting supernatants were used for measuring biochemical indices.

### 2.5. Statistical Analysis

To assess the effects of ZnO NPs on the tissues from each treatment group, the experimental data underwent an analysis via a three-way ANOVA. This analysis allowed for the evaluation of interactions among ZnO NPs, salinity, and exposure time within the treatments. In the event of significant interactions, differences between treatments were determined using a one-way ANOVA, followed by Tukey’s multiple comparison test. The resulting values, including the data presented in the Methods and Materials section, were expressed as mean ± standard error (SE) values, and the significance level for the tests was set at *p* ≤ 0.05. The data analysis was performed using SigmaPlot 11.0 (St. Louis, MO, USA).

## 3. Results

### 3.1. Characterization of ZnO NPs

The particle size distribution changed with time in the ZnO NP stock solution and gradually became stable. The average particle size of the ZnO NPs in water just after ultrasonic configuration was mainly concentrated around 650 nm. With an increase in the standing time, the change in the average particle size was not significant, remaining around 500 nm. The Zeta potential values of the medium at different time points fluctuated from −7.65 to −14.66 mV (Table 1).

During the settling process of the sample after ultrasonication (0–1 h), the Zeta potential values fluctuated from −7.65 to −10.09 mV. The actual Zn concentrations were 0, 3.699 ± 0.302, 5.914 ± 0.131, 8.235 ± 0.013, 9.934 ± 0.052, and 10.917 ± 0.096 mg L^−1^, respectively, in the six designed ZnO NP treatments (0, 10, 20, 40, 60, and 80 mg L^−1^).

### 3.2. Toxic Effects of ZnO NPs on Hatching and Larvae

**Figure 1:**
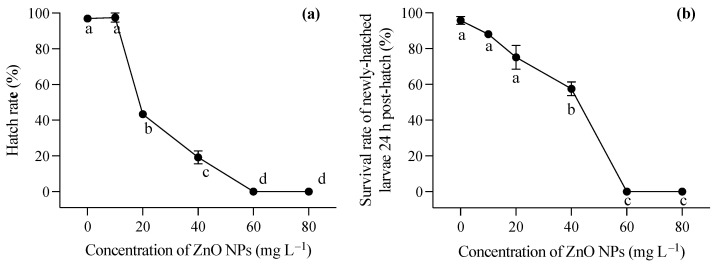
Hatching rate of *T. obscurus* embryos (**a**) and survival rate of *T. obscurus* larvae 24 h post hatch (**b**) under different ZnO NPs concentrations. Different letters represent significant differences (*p* < 0.05) among different ZnO NP concentrations.

During the embryonic development stage, the presence of ZnO NPs resulted in a coating of the embryo surface, causing hypoxia of the embryo and impeding the normal hatching process of larvae. As illustrated in Figure 1a, the hatching rate of embryos experienced a significant decrease with an increase in ZnO NP concentration (one-way ANOVA, *F* = 509.012, *p* < 0.001). In the lowest ZnO NP concentration group (10 mg L^−1^), there was no significant decrease in the hatching rate of embryos compared to the control group. However, when the concentration of ZnO NPs reached 20 mg L^−1^, the hatching rate dropped below 50%. At the highest two concentrations, fertilized eggs were unable to complete normal development, and hatching could not occur. The larvae, following successful hatching, were strongly impacted by exposure to ZnO nanoparticles within the first 24 h (*F* = 16.387, *p* = 0.006). Furthermore, the newly hatched larvae exhibited deformities in these conditions. Figure 1b demonstrated the survival rate of juveniles 24 h after hatching, indicating that higher ZnO NP contents contributed to a diminished survival rate, aligning with the observed hatching rate findings.

**Figure 2:**
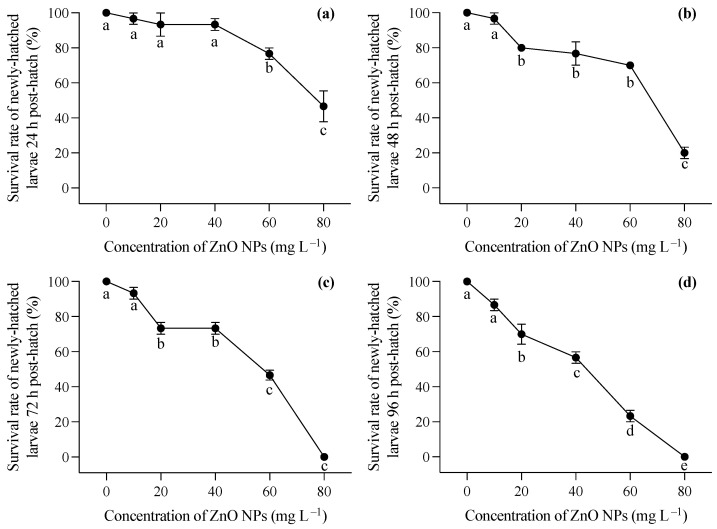
Survival rates at 24 h (**a**), 48 h (**b**), 72 h (**c**), and 96 (**d**) of *T. obscurus* larvae hatched in medium without ZnO NPs and subsequently exposed to different concentrations of ZnO NPs post hatching. Different letters represent significant differences (*p* < 0.05) among different ZnO NP concentrations.

**Figure 3:**
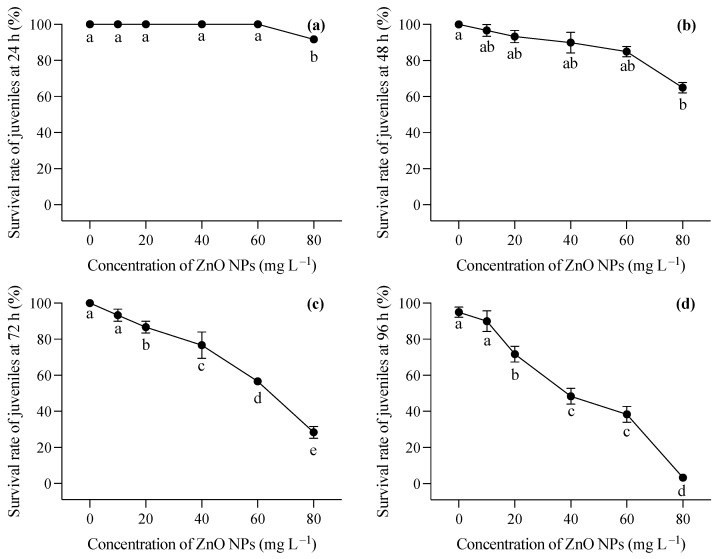
Survival rates at 24 h (**a**), 48 h (**b**), 72 h (**c**), and 96 (**d**) of *T. obscurus* juveniles exposed to different concentrations of ZnO NPs. Different letters represent significant differences (*p* < 0.05) among different ZnO NP concentrations.

Larvae hatched in a medium free of ZnO NP contamination were immediately exposed to varying concentrations of ZnO nanoparticles after hatching. Generally, increasing concentrations of ZnO NPs post hatching had a significant impact on the survival rates of *T. obscurus* larvae at all time points (one-way ANOVA, *p* < 0.05), with the effects becoming more pronounced over time. In contrast to the larvae hatched in ZnO NP-contaminated conditions, those hatched in ZnO NP-free conditions exhibited significantly lower mortality rates, as evident in the comparison between Figure 1b and Figure 2a. Initially, at 0 mg L^−1^ of ZnO NPs, the survival rate remained consistently high at 100% across all time intervals (Figure 3). As the concentration of ZnO NPs increased, a gradual decline in survival rates became apparent. At 10 mg L^−1^, there was a slight decrease in survival over time. Notably, the steepest decline occurred at 80 mg L^−1^, at which the survival rate dropped significantly, particularly at 72 h and 96 h (Figure 2c,d). After 72 h, less than half of the larvae survived under 60 mg L^−1^ ZnO NPs, and all individuals died at 80 mg L^−1^ (Figure 2c,d).

### 3.3. Toxic Effects of ZnO NPs on Juveniles

The changing trend in the survival rate of the two-month-old *T. obscurus* juveniles under different concentrations of ZnO NPs reveals a notable pattern. At the lowest ZnO concentration (0 mg L^−1^), the juveniles exhibited a consistent 100% survival rate across all time points (Figure 3). As the ZnO concentration increased, there was a general trend of decreasing survival rates over time. Higher concentrations of ZnO NPs (40, 60, 80 mg L^−1^) corresponded to a more pronounced decline in survival rates, with a significant drop observed at 96 h (Figure 3). During the first 24 h, mortality was only observed under 80 mg L^−1^ of ZnO NPs (Figure 3a), whereas death happened in all ZnO NP treatments at 48 h, and a significant decrease in the survival rate was shown at the highest ZnO NP concentration (Figure 3b). After 72 h, the survival rate of the larvae showed a progressive reduction with the increase in the ZnO NP concentration (Figure 3c,d). Notably, over 50% of the larvae perished after 96 h of exposure to ZnO NPs exceeding 40 mg L^−1^ (Figure 3d).

**Figure 4:**
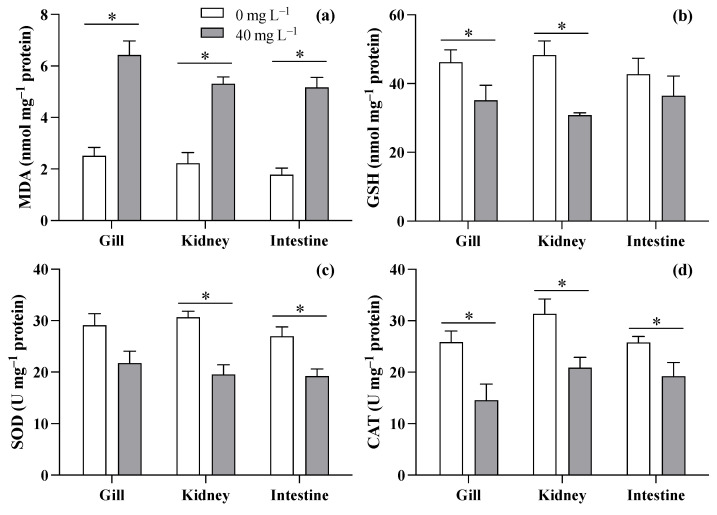
MDA (**a**), GSH (**b**), SOD (**c**), and CAT (**d**) in three tissues of *T. obscurus* juveniles exposed to 0 and 40 mg/L of ZnO NPs at 96 h. The asterisks (*) above the black bars denote a significant difference between the two ZnO NP concentrations (*p* ≤ 0.05).

Based on the survival experiment results, this study discusses the biochemical responses of the gill, kidney, and intestine of *T. obscurus* juveniles to ZnO NPs at 40 mg L^−1^ compared to 0 mg L^−1^. The results showed increases in MDA levels across all three tissues (gill: *F* = 37.11, *p* = 0.004; kidney: *F* = 39.236, *p* = 0.003; intestine: *F* = 37.11, *p* = 0.004) when exposed to ZnO NPs at 40 mg L^−1^ compared to the control (Figure 4a). As for GSH (Figure 4b), 40 mg L^−1^ of ZnO NPs caused significant decreases in the gill (*F* = 11.685, *p* = 0.027) and kidney (*F* = 23.16, *p* = 0.008), but no significant difference was observed in the intestine (*F* = 11.685, *p* = 0.027). Though reductions in SOD activity were observed in all three tissues (Figure 4c), significant differences were only observed in the kidney and intestine (gill: *F* = 5.127, *p* = 0.086; kidney: *F* = 25.900, *p* = 0.007; intestine: *F* = 11.150, *p* = 0.029). The exposure to ZnO NPs at 40 mg L^−1^ generally led to a reduction in CAT levels across all three tissues of *T. obscurus* juveniles compared to the control (gill: *F* = 26.694, *p* = 0.007; kidney: *F* = 26.390, *p* = 0.007; intestine: *F* = 15.178, *p* = 0.018).

## 4. Discussion

ZnO NPs are extensively used in various applications, and the small size and large surface area of ZnO NPs contribute to their increased mobility and surface activity, posing potential harm to organisms and environments. This study demonstrated that ZnO NPs negatively affected the development of the early life stages (hatching, larval, and juvenile) of *T. obscurus*.

ZnO NP exposure during the hatching stage resulted in a significant decrease in hatching rates, with embryos displaying surface coatings at higher ZnO NP concentrations. The embryo stage, as the most susceptible in the life cycle, is highly prone to diverse environmental stresses [25]. In the present study, the concentration of ZnO NPs demonstrated a direct correlation with the inhibition of embryo incubation and the mortality of newly hatched larvae, with no surviving embryos observed when exposed to ZnO NP concentrations exceeding 60 mg L^−1^ (Figure 1). Hatching retardation caused by ZnO NPs could be attributed to hypoxia due to the coverage of ZnO NPs on the embryos and the disturbance in hatching enzyme activity [26,27]. Valdiglesias et al. reported that the exposure to ZnO NPs of zebrafish embryos commonly led to decreased hatching rates, which might be attributed to a disturbance in hatching enzymes, particularly Zn-metalloproteases secreted by the embryo’s hatching gland, and a parallel trend was observed with ZnSO_4_ treatments, suggesting that the released Zn^2+^ ions, particularly at 100 mg L^−1^, may contribute to the observed reduction in hatching rates caused by ZnO NP exposure [28]. However, Hua et al. investigated the toxicity of different shapes of ZnO NPs to zebrafish embryos, revealing that ZnO NPs in each suspension exhibited higher toxicity than dissolved Zn^2+^ [29]. Additionally, Zn^2+^ dissolution enhanced the toxic effects of ZnO NPs to some extent, while filtered pure nanoparticles can induce oxidative stress, DNA damage, and developmental toxicity in embryos [30]. The solubility of ZnO nanoparticles (NPs) in a medium exhibited a dependency on the initial concentration, with the formation of larger aggregations observed at higher concentrations. This phenomenon can be attributed to the decreased surface-to-volume ratio inherent in larger aggregations, leading to a reduced release of ions [31]. In our study, the surfaces of the embryos were covered by nanoparticles, which may be partly responsible for the delayed development and hatching failure of the embryos, in addition to the toxicity caused by dissolved Zn ions. Additionally, particles with sizes exceeding 50 nm were mainly adsorbed onto the intestinal tract and outer epidermis of the zebrafish embryos [32]. Hence, beyond the direct toxicity induced by dissolved Zn ions, the incorporation of ZnO nanoparticles into embryos resulted in enduring impacts on both embryo development and hatching, along with the subsequent survival of newly hatched larvae.

Newly hatched larvae from ZnO NP-contaminated conditions experienced malformation, and post-hatching exposure leads to pronounced reductions in survival rates, particularly with higher ZnO NP concentrations. Unlike the larvae hatched under ZnO NP-contaminated conditions, those hatched in ZnO NP-free conditions displayed markedly lower mortality rates (Figure 1b and Figure 2a). The occurrence of malformation in newly hatched larvae exposed to ZnO NPs was consistent with previous studies [27,33]. These malformations encompassed fin rot, spinal deformities, craniofacial deformities, pericardial edema, yolk sac edema, and stretched heart. The most obvious malformations observed in our study were spinal deformities and pericardial and yolk sac edemas, which was consistent with findings in fish embryos exposed to various nanoparticles in previous studies [30,33,34]. The absorption of ZnO NPs in newly hatched larvae primarily relies on surface pathways due to the underdeveloped respiratory system and incomplete digestive tract in the early stages. Nanoparticles may adhere to the skin or be taken up, contributing to their internalization. The extensive dispersion of ZnO nanoparticles in natural water bodies and their subsequent sediments pose significant environmental risks. In Laizhou Bay, Yellow Sea, China, four metal-based NPs (Ti, Zn, Ag, Cu) were detected at all surface water stations, with the concentration of Zn-based NPs ranging from 0.60 × 10^7^ to 1.27 × 10^8^ particles L^−1^ [35]. In our study, a time-dependent toxicity of ZnO NPs to newly hatched larvae existed, and increased mortality and malformations were even observed at the lowest ZnO NP level (10 mg L^−1^). Our results indicate the potential for long-term impacts on development and survival even at environmentally relevant concentrations.

Two-month-old juveniles exposed to increasing ZnO NP concentrations exhibited a consistent decline in survival rates, emphasizing concentration-dependent adverse effects. In comparison with the survival of newly hatched larvae, juveniles were less sensitive to ZnO NPs (Figure 3). The juveniles might uptake ZnO NPs through the epidermis and gill as well as the intestine. It is crucial to closely monitor organ uptake and internal distribution for a more comprehensive understanding of NPs’ toxicity [32]. When absorbed, ZnO NPs would affect juveniles due to the toxicity of zinc ions released from the particles, causing stress responses in the fish [36]. Biochemical analyses revealed elevated MDA levels and decreased GSH contents, SOD activity, and CAT activity in the gill, kidney, and intestine, indicating oxidative stress caused by ZnO NPs. Elevated levels of MDA, a marker of lipid peroxidation, suggest increased oxidative damage to cellular membranes in these tissues. The induction of oxidative damage emerged as a primary toxic mechanism of TiO_2_ NPs in aquatic organisms such as fish and bivalves [24,37]. A study on the toxic effects of polystyrene nanoplastics showed that carp exposed to 1000 μg L^−1^ of PS-NPs of different sizes exhibited increased ROS levels, reduced antioxidant enzyme activities (CAT, SOD1, and Gpx1), and accumulated MDA over a 28-day period [38], suggesting that severe oxidative damage could inhibit the activity of antioxidant enzymes. Bobori et al. found that exposure to TiO_2_ nanoparticles induced oxidative stress in the gills and liver of adult zebrafish, indicating that gill damage and hypoxia may contribute to a reduction in swimming speed in fish [39]. Similarly, high oxidative stress in the gills was observed in the *T. obscurus* exposed to ZnO NPs in our study, which could lead to hypoxia in fish. In addition, ZnO NP exposure can potentially cause malformations in various fish organs. For instance, significant malformations of the olfactory rosettes at histological, ultrastructural, and genetic levels happened after chronic exposure to ZnO NPs [40]. ZnO NPs ingested during the feeding process have been found to impact intestinal structure and microbial abundance, thereby influencing glucose and lipid metabolism in fish [41]), suggesting severe functional alternations in the intestine caused by nanoparticle contamination. Therefore, future research will focus more on investigating the impacts of ZnO NPs on the functions of various organs in fish.

## 5. Conclusions

Consequently, the substantial decrease in the hatching rate of embryos and the survival rate of larvae and juveniles of *T. obscurus* observed in this study with an increasing ZnO NP concentration can be attributed primarily to the toxicity of nanoparticles, with Zn^2+^ playing an auxiliary role. High oxidative stress was induced by ZnO NPs in various tissues. This study underscores the ecological risks of ZnO NP contamination in aquatic environments, emphasizing the need for careful consideration of nanoparticle exposure in aquatic ecosystems.

## Figures and Tables

**Table 1:** The particle size and Zeta potential of ZnO NPs in water.

	0 h	1 h	6 h	12 h	24 h
Particle size (nm)	650.6 ± 21.70	576.67 ± 9.85	563.13 ± 6.06	535.79 ± 10.01	503.77 ± 11.65
Zeta potential (mV)	−7.63 ± 0.13	−10.24 ± 0.11	−14.13 ± 0.35	−13.97 ± 0.17	−13.26 ± 0.33

## Data Availability

The data presented in this study are available on request from the corresponding author.
